# Morphological Responses of Excitatory Prelimbic and Orbitofrontal Cortical Neurons to Excess Corticosterone in Adolescence and Acute Stress in Adulthood

**DOI:** 10.3389/fnana.2020.00045

**Published:** 2020-09-08

**Authors:** Elizabeth T. Barfield, Michelle K. Sequeira, Ryan G. Parsons, Shannon L. Gourley

**Affiliations:** ^1^Departments of Pediatrics and Psychiatry, Emory University School of Medicine, Yerkes National Primate Research Center, Emory University, Atlanta, GA, United States; ^2^Graduate Training Programs in Neuroscience, Emory University, Atlanta, GA, United States; ^3^Graduate Program in Integrative Neuroscience, Department of Psychology, Stony Brook University, Stony Brook, NY, United States

**Keywords:** metaplasticity, orbital, spine, stress, mPFC, oPFC

## Abstract

Considerable evidence indicates that chronic stress and excess glucocorticoids induce neuronal remodeling in prefrontal cortical (PFC) regions. Adolescence is also characterized by a structural reorganization of PFC neurons, yet interactions between stress- and age-related structural plasticity are still being determined. We quantified dendritic spine densities on apical dendrites of excitatory neurons in the medial prefrontal cortex, prelimbic subregion (PL). Densities decreased across adolescent development, as expected, and spine volume increased. Unexpectedly, exposure to excess corticosterone (CORT) throughout adolescence did not cause additional dendritic spine loss detectable in adulthood. As a positive control dendrite population expected to be sensitive to CORT, we imaged neurons in the orbitofrontal cortex (OFC), confirming CORT-induced dendritic spine attrition on basal arbors of layer V neurons. We next assessed the effects of acute, mild stress in adulthood: On PL neurons, an acute stressor increased the density of mature, mushroom-shaped spines. Meanwhile, on OFC neurons, dendritic spine volumes and lengths were lower in mice exposed to both CORT and an acute stressor (also referred to as a “double hit”). In sum, prolonged exposure to excess glucocorticoids during adolescence can have morphological and also metaplastic consequences, but they are not global. Anatomical considerations are discussed.

## Introduction

Glucocorticoids (primarily corticosterone in rodents) are principal mediators of the stress response. They mobilize energy stores to enable organisms to cope with stress, and are potent modulators of synaptic plasticity, with precise neurobehavioral effects varying depending on the intensity, duration, context, and developmental timing of stressor exposure. For example, mild, acute stress causes synaptic plasticity in the prefrontal cortex (PFC) and improves certain cognitive functions (Joëls et al., 2006; Popoli et al., 2011; McEwen and Morrison, 2013)—effects generally considered adaptive. Meanwhile, chronic stress or prolonged exposure to elevated glucocorticoids extensively reorganizes PFC neuronal architecture (Leuner and Shors, 2013; McEwen and Morrison, 2013). Further, the degree of stress-related disruptions in PFC-dependent functions (attention, working memory, goal-directed decision making) in some cases correlates with the degree of neuronal structural change in PFC regions (Liston et al., 2006; Hains et al., 2009; Barfield and Gourley, 2019). Chronic stress can also impact synaptic plasticity in response to subsequent experiences, an example of stress-induced metaplasticity (Luczynski et al., 2015; Nasca et al., 2015).

The PFC is among the last brain regions to structurally mature (Giedd et al., 1999; Gogtay et al., 2004). Following an initial phase of dendritic spinogenesis during childhood, a protracted period of spine elimination occurs during adolescence and into young adulthood (Petanjek et al., 2011). Such dramatic structural remodeling during adolescence may open a window of vulnerability to insults like chronic stress. Although the effects of a chronic stressor or glucocorticoid exposure during adulthood—such as dendritic spine attrition in the PFC—have been well characterized (McEwen and Morrison, 2013; Qiao et al., 2016), much less is known regarding the response of still-developing adolescent neurons. Initial studies reported patterns of dendritic spine loss in the PFC following repeated stress or excess glucocorticoids in adolescence (Gourley et al., 2013; Barfield et al., 2017; Pinzón-Parra et al., 2019), with measures largely collected in adolescence. What remains unclear is whether these modifications impact PFC maturational trajectories. Here, we exposed mice to excess CORT during adolescence and measured dendritic spine densities and morphologies on apical dendrites in the prelimbic medial PFC (PL) at several points throughout postnatal development. Our observations unexpectedly reveal apparent resiliencies in the PL.

Given our unexpected findings, we next imaged a distinct dendrite population that we anticipated to be sensitive to excess CORT in adolescence—excitatory neurons in the lateral orbitofrontal cortex (OFC). Here, CORT caused spine attrition, replicating prior investigations (Gourley et al., 2013; Barfield and Gourley, 2019).

## Materials and Methods

### Subjects

Group-housed male and female mice expressing *Thy1*-derived yellow fluorescent protein (YFP; H line from Feng et al., 2000) were back-crossed onto a C57BL/6 background. Mice were housed 2–5/cage and provided a 12-h light cycle (08:00 on) and food and water *ad libitum*. Procedures were approved by the Emory University IACUC and carried out following the recommendations of the *Guide for the Care and Use of Laboratory Animals*.

### CORT Exposure

4-pregnen-11β 21-DIOL-3 20-DIONE 21-hemisuccinate (Steraloids) was dissolved in tap water (25 μg/ml free base; Gourley et al., 2008a,b; Barfield et al., 2017). CORT-exposed mice were given CORT-infused drinking water, while control mice consumed tap water.

Water bottles were weighed daily, and mice weighed every other day, allowing us to calculate the amount of liquid displaced/total weight of all mice in the cage. With this value and the known concentration of CORT in hand, we were then able to calculate the approximate dose of CORT ingested by a mouse in any given cage (5–9 mg/kg/day). Every 3 days, water bottles were refilled with freshwater or newly prepared CORT solution.

**Figure 1:**
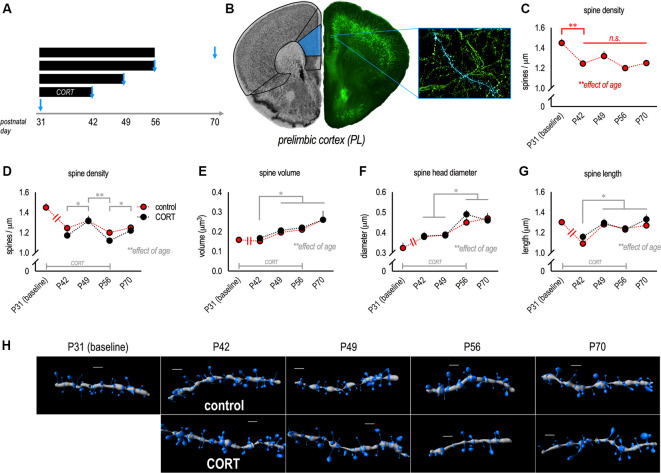
Dendritic spine densities in the prelimbic subregion (PL) decline during adolescence. **(A)** Experimental timeline of corticosterone (CORT) exposure and points of euthanasia. **(B)** Neurons were imaged from the PL. **(C)** Gross dendritic spine densities in control mice dropped from P31 to P42. Note that symbols represent independent groups of mice, but are connected to highlight patterns of change over time. **(D)** Next, we compared control and CORT-exposed mice, revealing additional age-dependent effects (though no effects of CORT). Dendritic spine densities transiently increased in late adolescence (P49), then dropped in young adulthood (P56) before modestly increasing again in adulthood (P70). **(E)** Dendritic spine volume increased over time. **(F)** Dendritic spine head diameters and **(G)** spine length also increased. **(H)** Representative PL dendrites reconstructed in 3D. Scale bars = 2 μm. Symbols represent means ± SEMs. *p ≤ 0.05, **p ≤ 0.001, n.s.: non-significant.

Mice were exposed to CORT or water from postnatal day (P) 31–56 to span most of the rodent adolescent period (Spear, 2000). Mice were euthanized before CORT exposure (P31, baseline), during CORT exposure (P42, P49, P56), or at P70 following a 2-week washout period without CORT (see the timeline in Figure 1A).

### Acute Stress Challenge

To determine whether chronic adolescent CORT exposure alters stress-induced structural plasticity, mice were subjected to a cage change, considered a common mild stressor (Rasmussen et al., 2011), ~3 h before euthanasia on P56. In this case, mice were transferred with cage mates to a clean cage containing fresh bedding. Meanwhile, control mice were undisturbed.

### Dendritic Spine Imaging and Reconstruction

Mice were briefly anesthetized by isoflurane and euthanized by rapid decapitation. Brains were submerged in chilled 4% paraformaldehyde for 48 h, then transferred to 30% w/v sucrose, and sectioned into 40–50 μm-thick sections on a freezing microtome. Dendritic segments on layer V pyramidal neurons in the PL, located between Bregma +1.98 to +1.7, were imaged with a spinning disk confocal (VisiTech International) on a Leica microscope. Z-stacks were taken with a 100× 1.4 numerical aperture objective using a 0.1 μm step size.

Seven to eight segments/mouse, collected from secondary and tertiary apical dendritic branches on independent neurons, 10–35 μm in length, within 50–150 μm of the soma, were imaged. We favored apical dendrites because basal dendrites appear to be more static during adolescent development by comparison (Mallya et al., 2019). Also, apical dendrites are considered highly stress-sensitive (Leuner and Shors, 2013).

For a positive control comparison, 4–7 segments/mouse, collected from independent neurons in the OFC were imaged in the same P56 mice. The sections were located between Bregma +2.8 and +2.22. Dendritic segments, 15–20 μm in length, within 50–150 μm of the soma, were imaged. We focused on basal arbors because dendritic spines on these arbors are sensitive exogenous CORT exposure in adolescence (Barfield and Gourley, 2019), as well as other adolescent experiences, including olanzapine treatment (Milstein et al., 2013), environmental enrichment (Mychasiuk et al., 2014), and cocaine exposure (DePoy et al., 2017).

Images were processed and dendritic spines reconstructed in 3D by an experimenter blind to the group using Imaris software (described Gourley et al., 2013). Morphological parameters based on those established by Radley et al. (2013) were used to classify spines as thin-, mushroom-, or stubby-type. These parameters were: thin (length >0.6 μm and ≤4 μm; head:neck ratio ≤1.5 μm), mushroom (length ≤4 μm; head:neck ratio ≥1.5 μm), or stubby (length ≤0.6 μm; head:neck ratio ≤1.5 μm).

Group (control/CORT) sizes were: P31 baseline (*n* = 4), P42 (*n* = 7/*n* = 7), P49 (*n* = 7/*n* = 8), P56 [no cage change (*n* = 9/*n* = 9) and cage change (*n* = 6/*n* = 7)], and P70 (*n* = 5/*n* = 4). Each mouse contributed a single density value (its average) to each comparison.

### Statistical Analyses

Two-tailed statistical analyses with *α* ≤ 0.05 were performed using SPSS. Dendritic spine density, volume, head diameter, and length were compared by 1-, 2-, or 3-factor analysis of variance (ANOVA), with age, CORT, or cage change as between-subjects factors, as appropriate. Following interactions or main effects of age, Tukey’s *post hoc* tests were applied, and results are indicated graphically. Spine morphometric measures were also compared by Kolmogorov–Smirnov (K–S) comparisons. Throughout, values ±2 standard deviations from the mean were considered outliers and excluded.

## Results

### Effects of Adolescent Development and CORT Excess on Excitatory PL Neurons

Here, we quantified the density and morphology of dendritic spines on apical dendrites of pyramidal neurons in the PL throughout adolescence and young adulthood in control and CORT-exposed mice (Figures 1A,B). Densities in control mice decreased across time, as expected (main effect *F*_(4,30)_ = 9.4, *p* < 0.001; Figure 1C). In particular, densities dropped between P31 and P42, consistent with prior reports (Shapiro et al., 2017). These and all other significant *post hoc* comparisons are indicated graphically.

Next, we compared densities and morphologies between control and CORT-exposed mice using a CORT exposure procedure that recapitulates the peak blood serum CORT levels induced by forced swim stress. We identified no effects on gross densities (no main effect *F*_(1,52)_ = 1.4, *p* = 0.2; no interaction *F* < 1; Figure 1D). Unexpectedly, with this larger population of mice, we detected a slight but significant elevation at P49 (late adolescence), suggesting that spine densities may transiently “rebound” during late adolescence (main effect of age *F*_(3,52)_ = 6.2, *p* = 0.001; Figure 1D).

As with dendritic spine densities, age but not CORT impacted dendritic spine morphology on apical PL dendrites: Spine volume increased over time (the main effect of age *F*_(3,53)_ = 8.5, *p* < 0.001; Figure 1E), without group differences (no main effect of CORT or interaction *F*’s < 1). Head diameters also increased (the main effect of age *F*_(3,53)_ = 12.9, *p* < 0.001; Figure 1F), with no effect of CORT (no main effect of CORT *F* < 1; no interaction *F*_(3,53)_ = 1.2, *p* = 0.3). Dendritic spine length increased with age (the main effect of age *F*_(3,53)_ = 8.0, *p* < 0.001; Figure 1G), also without group differences (no main effect of CORT or interaction *F*’s < 1). Representative reconstructed dendritic segments are shown in Figure 1H.

**Figure 2:**
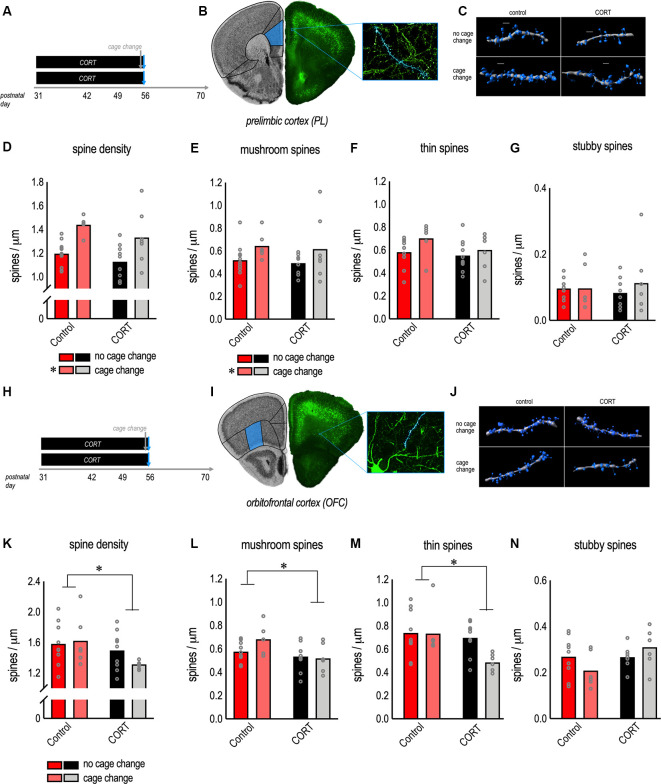
Morphological response of PL and orbitofrontal cortex (OFC) neurons to excess CORT in adolescence.** (A)** Experimental timeline and groups. **(B)** *Thy1*-YFP-expressing neurons from the PL. **(C)** Representative reconstructed apical dendrites. Scale bars = 2 μm. **(D)** Chronic CORT had no effects, while cage change increased gross dendritic spine densities. **(E)** Cage change triggered mature mushroom-shaped dendritic spines in the PL, a change that was unaffected by prior chronic CORT exposure. **(F)** Thin-type spines were unaffected. **(G)** Stubby spines were unaffected. **(H,I)** Pyramidal neurons in the lateral OFC were imaged from the same mice as a positive control for the effects of CORT. **(J)** Representative reconstructed dendrites (basal). Scale bars = 3 μm.** (K)** Chronic CORT reduced gross dendritic spine densities and **(L)** the density of mushroom-shaped dendritic spines. **(M)** Chronic CORT also reduced thin-type spine densities in the OFC. **(N)** Stubby spines were not significantly affected. Bars represent means, and data points represent individual mice. *p < 0.05.

To further explore possible effects of CORT, we focused on the P56 time point, the end of the CORT exposure period, and a period corresponding to young adulthood (Spear, 2000). We investigated whether CORT impacted experience-induced structural remodeling of apical dendrites on PL neurons, exposing some mice to a cage change (considered a common mild stressor) before euthanasia (Figures 2A–C). Cage change increased spine densities overall (the main effect of cage change *F*_(1,30)_ = 19.5, *p* < 0.001; Figure 2D), consistent with acute stress response (Nava et al., 2017). We did not detect a main effect of CORT (*F*_(1,30)_ = 3.0, *p* = 0.09; Figure 2D), nor a significant interaction between CORT and cage change (interaction *F* < 1; Figure 2D).

To further dissect these patterns, we classified spine subtypes. A primary finding was that cage change increased the density of mushroom-shaped dendritic spines in control and CORT-exposed mice (the main effect of cage change *F*_(1,30)_ = 4.6, *p* = 0.04; Figure 2E). We found no effect of CORT or interactions (*F*’s < 1; Figure 2F). When we analyzed thin-type dendritic spines, we found no effect of CORT (*F*_(1,30)_ = 1.9, *p* = 0.18; Figure 2F) or cage change (*F*_(1,30)_ = 3.3, *p* = 0.08; Figure 2F) or their interaction (*F* < 1; Figure 2F). For stubby-type dendritic spines, we again found no effect of CORT, cage change, or their interaction (all *F*’s < 1; Figure 2G). Thus, acute stress-induced dendritic spinogenesis appears to be attributable to mushroom-shaped dendritic spines.

### Excess CORT Causes Structural Plasticity in the OFC

The null effects of adolescent CORT exposure on apical dendrites in the PL were unexpected. As a positive control, we imaged pyramidal neurons in the OFC in the same mice (Figures 2H–J), focusing on basal dendrites. CORT exposure from P31 to P56 reduced gross dendritic spine densities in the OFC (the main effect of CORT *F*_(1,27)_ = 4.6, *p* = 0.04; Figure 2K). Cage change did not impact gross dendritic spine densities (the main effect of cage change *F* < 1; interaction *F*_(1,27)_ = 1.5, *p* = 0.24; Figure 2K).

Dendritic spine classification further defined the effects of CORT excess. Specifically, CORT caused attrition of mushroom-type spines in the OFC (the main effect of CORT *F*_(1,27)_ = 6.3, *p* = 0.02; Figure 2L), with no effect of cage change or their interaction (main effect of cage change *F*_(1,27)_ = 1.3, *p* = 0.27; interaction *F*_(1,27)_ = 2.1, *p* = 0.16; Figure 2L). CORT also reduced thin-type dendritic spine densities in the OFC (the main effect of CORT *F*_(1,27)_ = 5.6, *p* = 0.03; Figure 2M), with no effects of cage change (the main effect of cage change *F*_(1,27)_ = 3.2, *p* = 0.09; interaction *F*_(1,27)_ = 2.8, *p* = 0.10; Figure 2M). Neither CORT, nor cage change, nor their interaction significantly impacted the densities of stubby-type dendritic spines (no main effect of CORT *F*_(1,27)_ = 3.5, *p* = 0.07; no main effect of cage change *F* < 1; no interaction *F*_(1,27)_ = 3.6, *p* = 0.07; Figure 2N).

**Figure 3:**
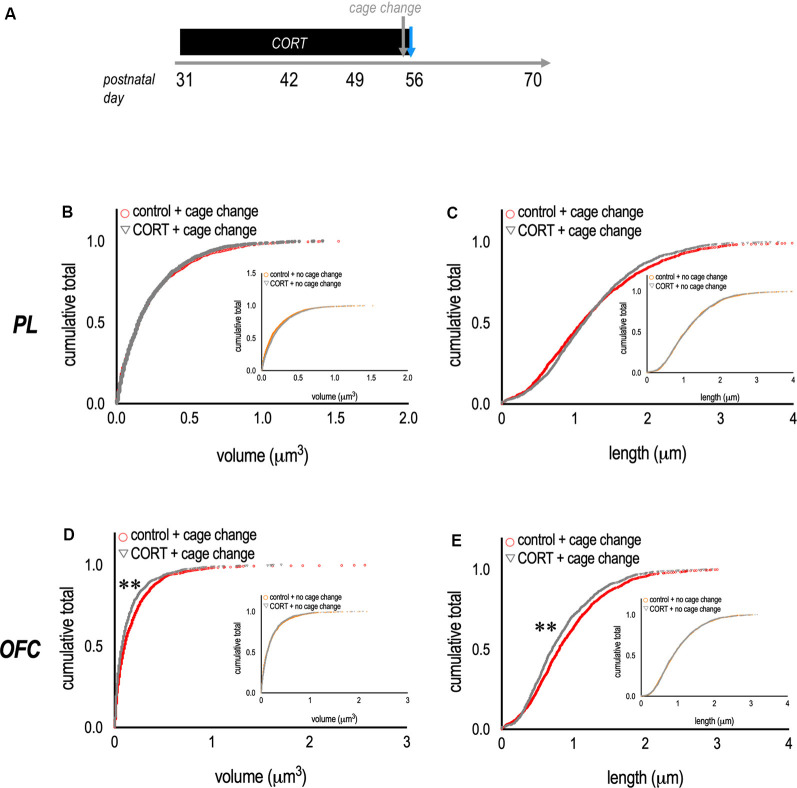
Metaplasticity in the OFC.** (A)** Experimental timeline and groups. **(B,C)** To complement data reported in the prior figure, we compared the volume and length of dendritic spine populations between groups. In mice with chronic CORT exposure and subjected to a cage change before euthanasia, dendritic spines in the PL appeared unaffected. **(D,E)** By contrast, mice with chronic CORT in adolescence and exposure to a cage change before euthanasia had a larger population of dendritic spines on OFC neurons with small volumes and short lengths—suggesting overall spine shrinkage. Inset: Effects could not be attributed to CORT alone. Each symbol represents a single dendritic spine. **p < 0.001.

Notably, the relative expression of thin-type dendritic spine densities is typically higher in PFC samples than those reported here, suggesting that some of the dendritic spines that were scored as mushroom-shaped may have been thin, or transitioning from thin to mushroom, and would be counted as thin spines using different classification parameters. Also notable, the pattern of dendritic spine densities in the OFC suggested that acute stress had subtle effects on thin-type dendritic spines, considered most vulnerable to stress (McEwen and Morrison, 2013). Another way to visualize dendritic spine morphologies is to assess the distribution of various spine parameters, agnostic to dendritic spine types. We compared dendritic spine morphometric measures between control + cage change vs. CORT + cage change mice (Figure 3A). Dendritic spine volumes and lengths in the PL did not differ between groups (K-S *p*’s > 0.1; Figures 3B,C). In contrast, spine volumes and lengths were lower in the OFC of CORT-exposed mice (K-S *p*’s < 0.001; Figures 3D,E), and these patterns were not attributable to CORT alone (figure insets). Thus, CORT systematically modified the morphological response of OFC neurons to an acute stressor.

## Discussion

Postnatal development is associated with the structural reorganization of neurons. The impact of stressors or glucocorticoid excess on these processes is still being defined. A prior experiment focused on a single time point in mid-adolescence and revealed that excess CORT caused dendritic spine attrition on the apical dendrites of PL neurons in mice (Barfield et al., 2017). Here, we aimed to determine whether excess glucocorticoids impacted the pattern of dendritic spine densities across time in the adolescent PL. Dendritic spine densities decreased as expected, while the average spine size became larger. To our surprise, CORT exposure did not affect these patterns, the ultimate distribution of dendritic spine subtypes in adulthood, or on the structural response of these neurons to an acute stressor. As a positive control dendrite population expected to be sensitive to CORT, we imaged basal dendrites on excitatory neurons in the nearby OFC, where CORT caused dendritic spine loss.

### The Density and Morphology of Dendritic Spines on PL Neurons Evolve Throughout Adolescence

In this investigation, we characterized dendritic spines on layer V PL neurons throughout adolescence and into adulthood. Gross dendritic spine densities on apical trees declined in early adolescence (between P31 and P42), consistent with prior reports (Pattwell et al., 2016; Shapiro et al., 2017; while basal arbors appear to be more static; see Mallya et al., 2019). Curiously, we found that gross dendritic spine densities transiently increased in late adolescence (P49). A similar spurt was identified in rats (Pinzón-Parra et al., 2019), though its significance remains unclear. Notably, *ex vivo* investigations like ours do not resolve whether spine attrition in adolescence is due to slowed spine formation rates, increased spine elimination rates, or a combination of these two factors. *In vivo*, two-photon microscopy can track individual spine formation and elimination rates, clarifying exact patterns (e.g., Johnson et al., 2016; Moda-Sava et al., 2019).

Dendritic spine volumes, head diameters, and lengths also increased across adolescence. Dendritic spine volume increased in a largely linear fashion from P31 to P70, while the largest increases in head diameter were evident between P49 and P56. Spine length also increased in mid-adolescence (P42–49), following an initial decrease in early adolescence. These patterns are reminiscent of other investigations in rodents and humans. Mallya et al. (2019) reported that dendritic spines in the rat PL at P39 were shorter, and had larger heads, compared to spines at P24, consistent with the initial decrease in spine length between P31 and P42 and progressive head enlargement found here. Further, Petanjek et al. (2011) observed that, in the human dorsolateral PFC, the heads of mushroom-shaped spines became slightly larger in late adolescence. Overall, these and other reports suggest that across species, dendritic spines on excitatory PFC neurons are pruned in adolescence, while remaining spines develop a more mature morphology, with larger heads capable of forming stable, functional synapses.

### Apparent Resistance to Excess CORT in the PL

Neuronal architecture in the medial PFC is highly sensitive to stress across the lifespan (McEwen and Morrison, 2013). In mature rodents, exogenous CORT exposure causes a loss of dendritic spines on apical dendrites of layer II, III, and V neurons in the PL (Swanson et al., 2013; Anderson et al., 2016). Nevertheless, we unexpectedly observed no consequences of excess CORT in adolescence. One factor might be that we focused on fairly caudal PL samples. This region projects to other mPFC structures (Heidbreder and Groenewegen, 2003) and receives projections from the hippocampus (Jay and Witter, 1991; Barbas et al., 1999). Meanwhile, the rostral PL is strongly connected with the amygdala (Heidbreder and Groenewegen, 2003; Vertes, 2004). Layer V neurons in the rostral PL suffer spine attrition following CORT exposure from P31 to P42 (Barfield et al., 2017), and social stressors during adolescence also cause dendritic spine loss on layer V PL neurons (Urban et al., 2019), though the rostrocaudal sampling site in this study was not defined. Future experiments could investigate what factors (anatomical, molecular, etc.) account for differences in rostral vs. caudal PL neurons. If certain dendritic spine populations are truly stress-resilient, it would be valuable to understand mechanisms. Further, what are the circuit-level consequences? For instance, might this pattern of dendritic spine attrition in the rostral PL vs. retention in the caudal PL lead to a prioritization of certain inputs to the PL at the expense of amygdalar inputs? If so, what are the impacts on cognitive and emotional functions?

Another factor to consider was our sampling scheme. We imaged several dendritic segments per mouse, within a defined distance from the cell soma, then generated a single average per mouse. While this strategy is not uncommon, it may be problematic. As elegantly reported by Katz et al. (2009) and others, synapse presence and structure can vary enormously along the dendritic tree, and thus, generating a single value per mouse likely under-samples and impoverishes what could be a richer characterization of dendrite morphologies. A more nuanced strategy may have revealed consequences of CORT that were over-looked here, particularly if it included distal dendritic tufts, which are highly stress-sensitive (McEwen and Morrison, 2013).

Repeated stress during periods of on-going brain maturation can modify molecular and cellular responses to subsequent stressors or CORT exposure (Eiland and McEwen, 2012; Majcher-Maślanka et al., 2018), examples of metaplasticity. We tested whether chronic CORT in adolescence had metaplastic consequences, but in the PL, we again detected no effects of CORT on dendritic spine densities. Instead, a mild cage change stressor (see Rasmussen et al., 2011) increased dendritic spine densities in the PL of control mice and mice exposed to CORT alike, consistent with prior investigations in rats (Nava et al., 2017). New dendritic spines were mushroom-shaped, suggesting that they were mature and functional—indeed, acute stress increases vesicular docking and synapse formation in the medial PFC (Nava et al., 2015). We speculate that acute stress-induced production of mushroom-shaped spines could provide synaptic connections necessary for a flexible strategy updating in response to threatening stimuli. Further, it appears likely that this response either habituates with repeated stress (since repeated CORT had few structural consequences for the same dendrites). Or, it is triggered by some aspect of the stress response other than CORT, such as NMDA receptor activation, given that NMDA receptor blockade obstructs stress-related dendrite remodeling on apical dendrites of excitatory neurons in the medial PFC (Martin and Wellman, 2011).

### CORT-Induced Structural Change in the OFC

Given the dearth of CORT-induced modifications of PL neurons, we imaged neurons in the OFC as a positive control. The OFC is often considered “prefrontal” and is sensitive to glucocorticoids. Excess CORT caused spine attrition on basal dendrites of OFC neurons, as expected. Specifically, CORT reduced the densities of mushroom- and thin-type dendritic spines, which could account for spine attrition in prior investigations that did not classify spine subtypes (Gourley et al., 2013). Notably, dendritic spine loss on OFC neurons also occurs with a shorter period of CORT exposure in adolescence (Barfield and Gourley, 2019) and parental neglect during a slightly earlier postnatal period (Helmeke et al., 2009)—multiple indications that adversities early in life can cause spine attrition on OFC neurons. Interestingly, the Kolb group recently exposed female rats to the routine presentation of novel cagemates, an experience that likely triggers a stress response. Dendritic spine densities on basal arbors of OFC neurons were reduced, as here (Hamilton et al., 2020). Finally, we also found that mice with CORT exposure + cage change just before euthanasia had small dendritic spines, an example of metaplasticity that could impair the signaling properties of these dendritic spines, given that large heads contain postsynaptic machinery necessary for optimal signal propagation, and long necks are thought to concentrate Ca^2+^ (Bourne and Harris, 2007).

One caveat regarding the use of cage change as a mild stressor is that animals of different ages and housed at different facilities will naturally have different cage change experiences, making it difficult to compare datasets across different institutions. For instance, more experience with cage change as a function of age will likely result in habituation of the stress response, even if other factors are comparable.

Why might OFC neurons be vulnerable to CORT? Under typical circumstances, intracellular signaling *via* the tropomyosin receptor kinase B (trkB, the high-affinity receptor for brain-derived neurotrophic factor; BDNF) facilitates the activity-dependent formation of nascent spines and the enlargement and stabilization of existing spines (reviewed Barfield and Gourley, 2018). Acute stress exposure increases the phosphorylation of trkB and downstream signaling proteins (Meller et al., 2003; Shen et al., 2004) in the rat PFC, but repeated stress or CORT down-regulates BDNF and trkB in the PFC (Gourley et al., 2009, 2012; Kutiyanawalla et al., 2011; Barfield et al., 2017; Zhang et al., 2017). Because these signaling proteins are more highly expressed in the OFC than mPFC (Shapiro et al., 2017), OFC neurons may be more sensitive to their loss upon excess CORT exposure.

Interestingly, repeated stress causes dendrite growth in the OFC (Liston et al., 2006; Dias-Ferreira et al., 2009). Acute stress in mice has similar consequences—stimulating the growth of apical OFC neuronal dendrites (Godar et al., 2015)—and routine cagemate change in rats causes growth on basal dendritic trees (Hamilton et al., 2020). These patterns are notable, given that mPFC neurons are more typically subject to stress-related dendrite loss (McEwen and Morrison, 2013). One consequence of dendritic arbor reorganization could be the biasing of inputs towards certain receptor systems. For example, 5-HT_2A_-mediated excitatory inputs are localized to apical dendrites on layer V neurons in the PFC (Liu and Aghajanian, 2008). Functional consequences of coincident stress-related dendrite growth and dendritic spine loss in the OFC remain unclear.

Importantly, in a prior report (Barfield et al., 2017), we confirmed that the CORT exposure procedure used here elevates blood serum CORT in adolescent mice to levels comparable to those following forced swim stress. It also causes adrenal and thymus gland atrophy that recovers when exogenous CORT is removed. And like chronic variable stress protocols, CORT exposure blunts body weight gain in adolescent mice (Barfield et al., 2017). The senior author and Jane Taylor initially developed this method (Gourley et al., 2008a,b) based on the seminal work of McEwen and colleagues focused on hippocampal neurons in rats (Magariños et al., 1998). Our concentration is notably lower—by 16-fold. We favor this “low” concentration because “low” and “high” concentrations have comparable effects in reducing sucrose consumption (a model for studying anhedonia in rodents) and degrading hippocampal neurotrophin systems in mice (Gourley et al., 2008a). Meanwhile, mice remain far healthier with lower concentrations.

## Conclusion

Stressors and stress hormones during adolescence remodel PFC neuron structure and synaptic density and induce deficits in PFC-dependent behaviors (Leussis et al., 2008; Gourley et al., 2013; Barfield et al., 2017; Pinzón-Parra et al., 2019). Our recent review of studies in rodents revealed that chronic stressors in adolescence need only be half as long in duration as chronic stressors in adulthood to produce comparable long-term behavioral impairments (Barfield and Gourley, 2018). Additionally, clinical evidence indicates that adverse experiences during childhood or adolescence are associated with negative psychiatric outcomes in adulthood (Martins et al., 2011; Carr et al., 2013). Here, we show that prolonged, excess glucocorticoids during adolescence have structural consequences detectable in adulthood in the OFC, while layer V neurons in the caudal PL are possibly resistant to these consequences. Future research should aim to identify how these differential sensitivities to adolescent CORT exposure might contribute to vulnerability (or resilience) to maladaptive effects of stress.

## Data Availability Statement

The datasets presented in this article are not readily available because data may be made available upon reasonable request. Requests to access the datasets should be directed to shannon.l.gourley@emory.edu.

## Ethics Statement

The animal study was reviewed and approved by Emory University Institutional Animal Care and Use Committee.

## Author Contributions

EB, MS, and RP conducted experiments and analyzed their data. EB prepared the manuscript. MS and SG revised the document.

## Conflict of Interest

The authors declare that the research was conducted in the absence of any commercial or financial relationships that could be construed as a potential conflict of interest.

The reviewer LD declared the author SG was previously their supervisor.
